# Evidence for Lignin Oxidation by the Giant Panda Fecal Microbiome

**DOI:** 10.1371/journal.pone.0050312

**Published:** 2012-11-28

**Authors:** Wei Fang, Zemin Fang, Peng Zhou, Fei Chang, Yuzhi Hong, Xuecheng Zhang, Hui Peng, Yazhong Xiao

**Affiliations:** 1 School of Life Sciences, Anhui University, Hefei, Anhui, China; 2 Anhui Provincial Engineering Technology Research Center of Microorganisms and Biocatalysis, Hefei, Anhui, China; University of Waterloo, Canada

## Abstract

The digestion of lignin and lignin-related phenolic compounds from bamboo by giant pandas has puzzled scientists because of the lack of lignin-degrading genes in the genome of the bamboo-feeding animals. We constructed a 16S rRNA gene library from the microorganisms derived from the giant panda feces to identify the possibility for the presence of potential lignin-degrading bacteria. Phylogenetic analysis showed that the phylotypes of the intestinal bacteria were affiliated with the phyla *Proteobacteria* (53%) and *Firmicutes* (47%). Two phylotypes were affiliated with the known lignin-degrading bacterium *Pseudomonas putida* and the mangrove forest bacteria. To test the hypothesis that microbes in the giant panda gut help degrade lignin, a metagenomic library of the intestinal bacteria was constructed and screened for clones that contained genes encoding laccase, a lignin-degrading related enzyme. A multicopper oxidase gene, designated as *lac51*, was identified from a metagenomic clone. Sequence analysis and copper content determination indicated that Lac51 is a laccase rather than a metallo-oxidase and may work outside its original host cell because it has a TAT-type signal peptide and a transmembrane segment at its N-terminus. Lac51 oxidizes a variety of lignin-related phenolic compounds, including syringaldazine, 2,6-dimethoxyphenol, ferulic acid, veratryl alcohol, guaiacol, and sinapinic acid at conditions that simulate the physiologic environment in giant panda intestines. Furthermore, in the presence of 2,2′-azino-bis(3-ethylbenzothiazoline-6-sulfonic acid) (ABTS), syringic acid, or ferulic acid as mediators, the oxidative ability of Lac51 on lignin was promoted. The absorbance of lignin at 445 nm decreased to 36% for ABTS, 51% for syringic acid, and 51% for ferulic acid after incubation for 10 h. Our findings demonstrate that the intestinal bacteria of giant pandas may facilitate the oxidation of lignin moieties, thereby clarifying the digestion of bamboo lignin by the animal.

## Introduction

The giant panda (*Ailuropoda melanoleuca*), of family *Ursidae* order *Carnivora*, is one of the most endangered animals in the world, with only 2,500 to 3,000 individuals found in western China [1]. Approximately 7 million years ago, the ancient giant panda was omnivorous, but it shifted from being an omnivore to a herbivore after 4.6 million years to 5 million years of evolution, with soft bamboo shoots, stems, and leaves comprising 99% of its diet [2], [3]. However, the modern giant panda retains a gastrointestinal tract typical of its carnivorous ancestry. The presence of a short straight colon and the absence of a cecum imply a digestive system more suitable for a meat diet than a bamboo diet. Furthermore, recent analysis of the giant panda genome revealed that it encodes all the enzymes necessary for a carnivorous digestive system but lacks those for digesting lignocellulose, which is the principal component of its bamboo diet [4]. Hence, the gut microbiome of giant pandas may play a key role in digesting fibrous plant materials because intestinal bacterial flora has been proven to significantly affect animal health and nutrient absorption [5].

Recently, several investigations have provided evidence for the presence of microbes in the giant panda intestines that help digest cellulose and hemicellulose. The putative genes responsible for cellulose and hemicellulose digestion (such as cellulase, β-glucosidase, xylan 1,4-β-xylosidase, and endo-1,4-β-xylanase) have been found in a metagenome of the gut microbes from three wild giant pandas [4]. However, cellulose and hemicellulose are usually embedded in a matrix of other structural biopolymers, primarily lignin, which comprises about 25% of bamboo dry weight [6]. Lignin is a major barrier during the enzymatic hydrolysis of cellulose. Removing lignin provides cellulase with easier access to cellulose [7]. As wood-feeding insects, termites are an excellent model system for studying lignocellulose digestion, and the idea that gut symbionts are important contributors to lignocellulose digestion is strongly supported in the literatures [8]–[10]. Thus, the gut microbes of giant panda are speculated to be responsible for the partial degradation of bamboo lignin during cellulose and hemicellulose utilization.

In nature, white-rot fungi are the major lignin degraders, which involve phenol oxidases (laccases) and peroxidases (lignin peroxidases and manganese peroxidases) as the key enzymes [11]–[14]. This enzymatic process exerted by bacteria is still unclear. However, several findings indicated that lignin is also degraded by bacterial enzyme systems such as the ones from *Comamonas* sp. B-9, *Pseudomonas putida* mt-2, and *Rhodococcus jostii* RHA1, from which extracellular laccases, as well as (or instead of) extracellular peroxidases from these bacteria were identified to help break down lignin [15], [16]. Laccases are multi-copper oxidoreductases that oxidize a variety of phenolic substances with or without redox mediators. In bacteria, the perceived roles of laccases have mostly been limited to the oxidation of metals and pigment formation. However, a growing number of studies suggest that bacterial laccases are important in lignin degradation alone or with other lignolysis-related enzymes [6], [17], [18].

Previous studies confirmed the presence of cellulose-digesting microbes associated with *Clostridium* clusters I and XIVa in the gut of giant pandas [4]. However, whether the gut microbes of the giant panda contribute to the lignin-related degradation remains unclear. In this study, we first identified the possibility for the presence of potential lignin-degrading bacteria in the giant panda gut based on 16S rRNA gene analysis. To validate the hypothesis that gut microbes help digest lignin, bacterial laccase, a lignolysis-related enzyme, was screened from the bacterial metagenomic library of giant panda gut. The bacterial laccase activity against phenolic lignin-related moieties and its effect on lignin with or without mediators was also evaluated. Our findings enhance our understanding of the mechanisms by which giant pandas utilize bamboo as an energy source, and the potential need for specific functional clades within the gut microbiome for proper giant panda development.

## Materials and Methods

### Ethics statement

Sample collection was conducted in the Hefei Safari Park, Anhui Province, China. Permission to conduct the research was granted by the park director and the Provincial Bureau of Forestry.

### Microorganisms and agents

*Escherichia coli* DH5α and *E. coli* BL21 (DE3) were obtained from TransGen (Beijing, China). *E. coli* EPI300 and the pIndigoBAC-5 vector were obtained from Epicentre (Madison, WI, USA). The pGEM-T vector was purchased from Promega (Madison, WI, USA). Syringaldazine, syringaldehyde, ABTS, 2,6-dimethoxyphenol, ferulic acid, veratryl alcohol, guaiacol, sinapinic acid, and lignin alkali were purchased from Sigma-Aldrich (St. Louis, MO, USA). All chemicals and reagents were of analytical grade.

### Sampling

Giant panda feces were collected from the Hefei Safari Park, Anhui, China, in February 2010. The feces were collected using sterile gloves immediately after defecation and were kept at −70°C until use. The sample used for bacterial DNA preparation was taken from the inside of the feces under sterile conditions.

### High-molecular-weight genomic DNA (HMW gDNA) extraction

The high-molecular-weight genomic DNA of the fecal microbes was prepared according to the methods by Walter et al. [19]. Briefly, the feces were diluted fourfold in ice-cold phosphate-buffered saline (pH 7.4) and shaken vigorously. The microbes in the feces were collected by gradient centrifugation and resuspended in 5 mL of STE buffer (0.2 M NaCl, 100 mM ethylenediaminetetra acetic acid (EDTA), 10 mM Tris, pH 8.0). Agarose plugs were made by mixing the cell suspension with the same volume of 1.5% low-melting-point agarose using a plug mold (Bio-Rad Laboratories, CA). The plugs were incubated at 37°C in 15 mL of lysis buffer (STE buffer containing 0.2% sodium deoxycholate, 1% sodium lauryl sarcosine, and 5 mg/mL of lysozyme). The plugs were then transferred into 15 mL of ESP buffer (1 mg/mL of proteinase K, 1% sodium lauryl sarcosine, 50 mM Tris, 0.5 M EDTA, pH 8.0) and incubated for 24 h at 55°C. Then, the plugs were transferred into 15 mL of fresh ESP buffer and incubated for another 24 h. The plugs were then transferred into 15 mL TE50 buffer (50 mM EDTA, 10 mM Tris, pH 8.0) containing 1 mM phenylmethylsulfonyl fluoride to inhibit proteinase K activity. After 2 h of incubation at room temperature, the plugs were washed four times with 5 mL of ice-cold TE50 buffer and then stored in this buffer at 4°C.

To efficiently remove the contaminants in the plugs that may partially inhibit digestion, the microbial DNA in the plugs was electroeluted into 1× Tris–acetate–EDTA (TAE) buffer [19], then concentrated on 0.025 µm membranes against 20% PEG8000, and stored in TAE buffer at 4°C for 16S rRNA gene library and metagenomic library construction.

### 16S rRNA gene library construction

Amplification of the 16S rRNA genes was performed using Bact-27F (AGAGTTTGATCMTGGCTCAG) and Univ-1492R (GGTTACCTTGTTACGACT) as primers [20], as well as HMW gDNA as template. The PCR products were ligated into pGEM-T vector. Positive clones were placed in 384-well plates to construct a 16S rRNA gene library.

The 16S rRNA gene sequences in the positive clones were amplified using the primer pair M13F (GTAAAACGACGGCCAG) and M13R (CAGGAAACAGCTATGAC). Then, the respective amplified products were fully digested with *Hae*III and *Hin*fI. The clones were then grouped based on the restriction fragment length polymorphisms (RFLP) of the digested DNA sequences. One to three clones in each group were selected as representatives for sequencing.

### Phylogenetic analysis

Putative chimeras were tested using the Mallard program [21]. The 16S rRNA gene sequences were classified using a classifier program from the Ribosomal Database Project [22]. The search for similar 16S rRNA gene sequences was performed using BLASTn from the National Center for Biotechnology Information (NCBI, http://www.ncbi.nlm.nih.gov/gorf/gorf.html). Sequences were assigned as operational taxonomic units (OTUs) using the Mothur program with 97% sequence similarity as the designated cutoff [23]. The phylogenetic tree was created using Clustal ×2.0 and MEGA 4.0 program.

The coverage of the 16S rRNA gene library was calculated using the formula [1-(n/N)], where n is the number of OTUs represented by one clone, and N is the total number of clones [24]. Bacterial diversity and richness were calculated using the Shannon–Weaver index [25]. Rarefaction analysis was performed using the Mothur program at 97% and 95% sequence similarity thresholds [23].

### Metagenomic library construction

Metagenomic library construction was performed according to Chu et al. [26]. Briefly, HMW gDNA was partially digested with 10 U *Bam*HI (New England Biolabs, Ipswich) for 6 min and separated by pulse field gel electrophoresis (CHEF-mapper, Bio-Rad). DNA fragments larger than 50 kb were cut from the gel, electroeluted into TAE buffer as mentioned above, concentrated on 0.025 µm membranes against 20% PEG8000, and then ligated into pIndigoBAC-5 (*Bam*HI cloning-ready, Epicentre) using T4 DNA ligase (Progema, USA). The ligation solution was then electrotransformed into the *E. coli* EPI300 competent cell. The transformed bacteria were plated on Luria-Bertani agar containing 12.5 µg/mL chloramphenicol, isopropyl-β-d-thiogalactopyranoside, and 5-bromo-4-chloro-3-indolyl-β-d-galactopyranoside. White colonies were picked and stored in 384-well plates at −70°C. The plasmids of white clones were randomly selected and digested by *Not*I to evaluate the insert size.

### Laccase-positive clone screening

Laccase-positive clone screening was performed based on both function-screening and sequence-screening strategies. When function-based methods were adopted, 1 mM syringaldazine or guaiacol was added into the agar plate. Clones surrounded by a purple or brownish red halo, which resulted from the hydrolysis of syringaldazine or guaiacol, were approved as positive clones [27], [28].

Sequence screening of laccase positive clones was performed according to the method of Fang et al. [29], using extracted plasmid DNA from white colonies as templates and the degenerate primer pair of the primers CuIF (ACMWCKGTTCAYTGGCACGG) and CuIVR (TGNTCNAGNAWGTGRCARTG), which were designed based on the conserved regions I and IV of copper-binding sites in bacterial laccases. Clones with a fragment of about 1.1 kb in the PCR products were considered as putative positive clones. Then, the 1.1 kb fragments were ligated into pGEM-T for sequencing.

Full-length laccase genes were obtained through inverse PCR with positive plasmids as templates. Briefly, plasmids extracted from the positive clones were fully digested with *Bam*HI, which has no cut site in the 1.1 kb fragment. The products were then self-ligated with T4 DNA ligase and used as a template using inverse primers of L51F (GCCATGCCAATGAATCGTGG) and L51R (ACTGCCATGTGATTGACCA), which were designed based on the 1.1 kb DNA information sequenced. The PCR products were ligated into pMD18-T vector (TaKaRa, Dalian, China) and sequenced.

### Sequence analysis of laccase

The open reading frame of *lac51* was determined using the Open Reading Frame Finder provided by NCBI. The modular structure of the enzyme was analyzed using SMART (http://smart.embl-heidelberg.de/). The presence and location of the signal peptides in the laccase sequence was predicted using the Neural Network and Hidden Markov models trained on Gram-negative bacteria with SignalP 3.0 program (http://www.cbs.dtu.dk/services/SignalP/) [30]. Multiple sequence alignment with other related laccase sequences was performed using Clustal ×2.0 and GeneDoc.

### Expression and characterization of Lac51

The putative laccase gene *lac51* obtained from the metagenomic library was cloned into pET22b (+) with and without its signal sequence and was heterologously expressed in *E. coli* BL21 (DE3). The pure enzyme was obtained using Ni^2+^-NTA affinity chromatography (Novagen, Darmstadt, Germany).

To evaluate the biochemical activity of Lac51, syringaldazine (ε_525_ = 65,000 M^−1^ cm^−1^) was used as substrate. The assay system contained 20 µL of appropriately diluted enzyme, 980 µL of 50 mM Na_2_HPO_4_-KH_2_PO_4_ (pH 7.5), 100 µM CuSO_4_, and 100 µM syringaldazine in a final volume of 1 mL. After incubation at 50°C for 5 min, the mixture was transferred into an ice-water bath for 30 s to stop the reaction, and the absorbance was measured at 525 nm. One activity unit (U) was defined as the amount of Lac51 required to oxidize 1 µmol of syringaldazine per minute. The alternative substrates of typical lignin-related phenolic compounds for laccase activity measurement were guaiacol (ε_465_ = 12,000 M^−1^ cm^−1^), sinapinic acid (ε_316_ = 8,360 M^−1^ cm^−1^), 2,6-dimethoxyphenol (ε_468_ = 49,600 M^−1^ cm^−1^), veratryl alcohol (ε_310_ = 9,000 M^−1^ cm^−1^), and ferulic acid (ε_318_ = 6,530 M^−1^ cm^−1^), at a final concentration of 1 mM. The heat-treated Lac51 was used as the control.

The effect of pH on laccase activity was determined using syringaldazine as the substrate using the following buffers: 50 mM sodium acetate buffer (pH 4.5 to pH 5.5), 50 mM Na_2_HPO_4_–KH_2_PO_4_ buffer (pH 5.5 to pH 8.0), and 50 mM Tris-HCl buffer (pH 8.0 to pH 9.0). The effect of temperature was measured from 15°C to 55°C in 50 mM Na_2_HPO_4_–KH_2_PO_4_ (pH 7.5).

### Protein and copper content determinations

The Lac51 protein concentration was routinely determined using the Bradford method with bovine serum albumin as the standard. The Cu content of the purified Lac51 was measured by atomic absorption spectroscopy according to Durão et al. [31].

### Enzymatic treatments of Lac51 towards lignin alkali

Lignin alkali was dissolved in 50 mM Na_2_HPO_4_–KH_2_PO_4_ buffer (pH 7.5) at a final concentration of 0.75 mg/mL. ABTS, guaiacol, ferulic acid, sinapinic acid, syringic acid, and syringaldehyde were used as mediators. The reaction mixtures contained 780 µL of 0.75 mg/mL lignin (pH 7.5), 100 µM CuSO_4_, and 200 µL of Lac51 (10 mU). Samples were incubated at 37°C for 10 h and monitored by full wavelength scanning from 200 nm to 800 nm. Additional assays were performed under the same conditions in the presence of 100 µM mediator.

### Nucleotide sequence accession numbers

The *lac51* and *lac9* nucleotide sequences were deposited to the GenBank database under accession numbers JN867369 and JQ082513, respectively. The corresponding Lac51 and Lac9 protein IDs were AEX55199 and AFD34359. The accession numbers for the 16S rRNA gene sequences were JN867371 to JN867373, JN867378 to JN867397, and JN867399 to JN867437.

## Results

### Potential lignin-degrading related bacteria exist in giant panda intestine

**Figure 1:**
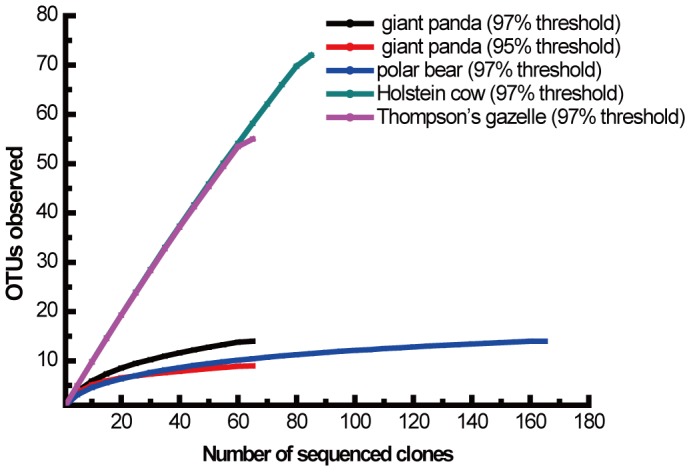
Rarefaction curves of 16S rRNA gene libraries recovered from the feces of giant panda and other mammalian gut microbial communities. The curves were generated based on analyses performed by the Mothur program. The expected number of clones was calculated from the number of clones analyzed at the species level with 97% sequence identity and genus level with 95% sequence identity. Data from polar bears (*Ursus maritimus*) are based on Glad et al [38]. Data from Holstein cows are based on Tajima et al [39]. Data from wild herbivore Thompson's gazelle (*Gazella rufifrons*) are based on Nelson et al [40].

To investigate the possibility for the presence of lignin-degrading related bacteria in the giant panda gut, 16S rRNA gene library was constructed using genomic DNA from fecal microbes as template. The 16S rRNA gene sequences of the 221 clones in the bacterial 16S rRNA gene library were randomly selected for RFLP analysis by fully digesting with *Hae*III and *Hin*fI. Based on the RFLP patterns, these sequences were classified into 25 OTUs. Among these sequences, 65 clones were further analyzed by sequencing. Except for 3 chimeras, 62 of the 16S rRNA gene sequences were classified into 14 different phylotypes with 97% sequence similarity as the designated cutoff (Table 1) [23]. Based on 97% sequence similarity, the coverage of the library was 90.3%, and the Shannon–Weaver index, which measures diversity, was 2.141. The rarefaction curve indicated that the number of clones analyzed was enough to investigate the bacterial community in the giant panda gut at either genus (95% sequence similarity) or species level (97% sequence similarity) (Figure 1) [32]. Reasonable coverage was obtained with cutoff of 97%, which indicated that the most abundant groups sampled were adequately covered.

**Table 1:** Identity values of 16S rRNA gene sequences retrieved from the intestinal bacteria of giant panda.

Phylotype	Accession number	No. of clones	Nearest relatives (Accession No)	Identity (%)
GP_38	JN867437	15	*Escherichia coli* (JN578646)	99
GP_B4	JN867384	2	*Escherichia coli* (JN547259)	98
GP_B47	JN867396	1	*Enterobacter cancerogenus* (JN644583)	97
GP_A8	JN867372	8	*Hafnia alvei* (AB244475)	99
GP_B6	JN867385	1	Uncultured bacterium (GQ451180)	96
GP_B55	JN867400	1	Uncultured *Pseudomonas* sp.(GU179631)	97
GP_B7	JN867386	1	Uncultured *gamma proteobacterium* (HM046581)	97
GP_B35	JN867392	3	*Pseudomonas putida* (AY450556)	99
GP_B40	JN867394	1	Uncultured *Methylobacterium* sp. (HQ674789)	99
GP_28	JN867431	4	Uncultured *Clostridium* sp. (GQ468578)	99
GP_83	JN867416	1	Uncultured *Clostridium* sp. (EU071533)	97
GP_53	JN867410	16	Uncultured *Clostridium* sp. (HQ132458)	99
GP_6	JN867423	4	Uncultured *Clostridium* sp. (HQ183774)	97
GP_8	JN867434	4	*Clostridium sartagoforme* (NR026490)	98

Of the 62 sequences, 47% were associated with class *Clostridia* of phylum *Firmicutes*. Four phylotypes (GP_6, GP_8, GP_53, and GP_83) representing 25 sequences were closely related to *Clostridium* group I and were branched with uncultured bacteria from the guts, feces, and composting bacteria (Figure S1). One phylotype that belonged to *Clostridium* group XI (4 sequences, 7% of the total sequences), was clustered with uncultured *Clostridium* sp. from polar bears, which is in the same order *Carnivora* as the giant panda.

The other 53% sequences were affiliated with phylum *Proteobacteria*, and the majority of the sequences (32 sequences, 52% of the total sequences) were affiliated with class *Gammaproteobacteria*. In this class, four phylotypes (26 clones) belonged to family *Enterobacteriaceae*, which were clustered with the bacteria pooled from the guts and feces of various animals such as sheep and human. Four phylotypes representing fifteen clones belonged to genus *Pseudomonas*. Of these, two phylotypes B_55 and B_35 were branched with the uncultured bacteria from mangrove forests, with 96% sequence identity. Mangrove forests are an important source of lignocellulose-degrading enzymes. A majority of fungi and bacteria from this type of habitat were shown to produce enzymes involved in biomass degradation including cellulase, xylanase, and laccase [27], [33], [34]. Furthermore, phylotype B_35 also showed 99% sequence identity to *P. putida*. Affiliated with the same species, *P. putida* mt2 has been proven to degrade aromatic compounds and lignin [15]. Thus, lignin degradation–related bacteria potentially inhabit the gut of giant pandas.

### Laccases exist in the intestinal microorganisms

To verify the aforementioned assumption that the gut microbes may participate in lignin degradation, a metagenomic library was constructed using the genome of giant panda fecal microbes and lignolysis-related genes were screened from the library. Based on blue-white screening strategy, about 98% of clones on plates were white clones. Approximately 31,000 white clones were picked out. Restriction endonuclease analysis (*Not*I) of 20 randomly chosen clones showed that the clones contained inserts between 20 kb and 150 kb in size with an average of 60 kb, which indicates that the library contained about 1.8 Gb genomic DNA information.

**Figure 2:**
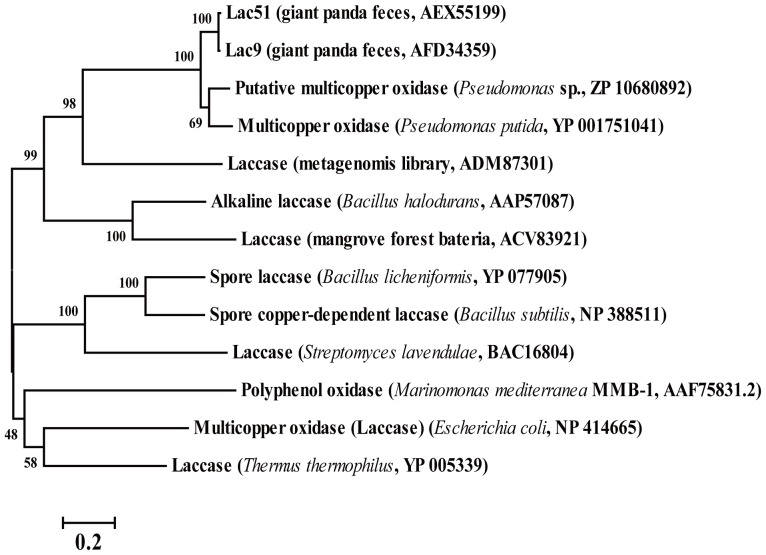
Laccase protein phylogeny. Phylogenetic tree was built based on an alignment of Lac51 and Lac9 with the nearest relative multicopper oxidases in *Pseudomonas* spp. and other representatives of bacteria from which laccase activity was demonstrated [18].

Bacterial laccases are important contributors during the bacterial lignin degradation process [6], [17], [18]. Consequently, laccase gene–containing clones were screened from the library. Based on the functional screening strategy, no positive clones with laccase activity were obtained. However, two bacterial multicopper oxidoreductase genes, namely, *lac9* and *lac51*, were screened using the sequence-based strategy with 99% sequence identity. Phylogenetic analysis showed that both Lac51 and Lac9 clustered with multicopper oxidases from *Pseudomonas* sp. (Figure 2), and *lac51* was chosen for further investigation.

The *lac51* ORF was 1,305 bp in length and encodes a 51 kDa polypeptide. The first 23 amino acid residues at the Lac51 N-terminal were identified as a TAT signal peptide (twin arginine translocation; Pfam: PF10518). A transmembrane segment was also predicted in Lac51, which ranged from the 7th to the 29th residue. A PSI-BLASTp search of NCBI database showed that Lac51 has the highest identity (88%) with the deduced amino acid sequence of a putative multicopper oxidase derived from *Pseudomonas* sp. (ZP_10680892), followed by the multicopper oxidases from *Pseudomonas entomophila* (YP_609858) and *P. putida* KT2440 (NP_743195). However, Lac51 shared low identity with typical bacterial laccases, with only 22% identity with copper-dependent laccase CotA (CAB12449) from *Bacillus subtilis* spores and 26% with *E. coli* CueO (BAB96698). Lac51 also has a low identity with laccases from uncultured bacteria, e.g., 33% identity with Lac15 from a marine microbial metagenome (ADM87301) and 23% with Lac591 from a mangrove soil metagenome (ACV83921).

**Figure 3:**
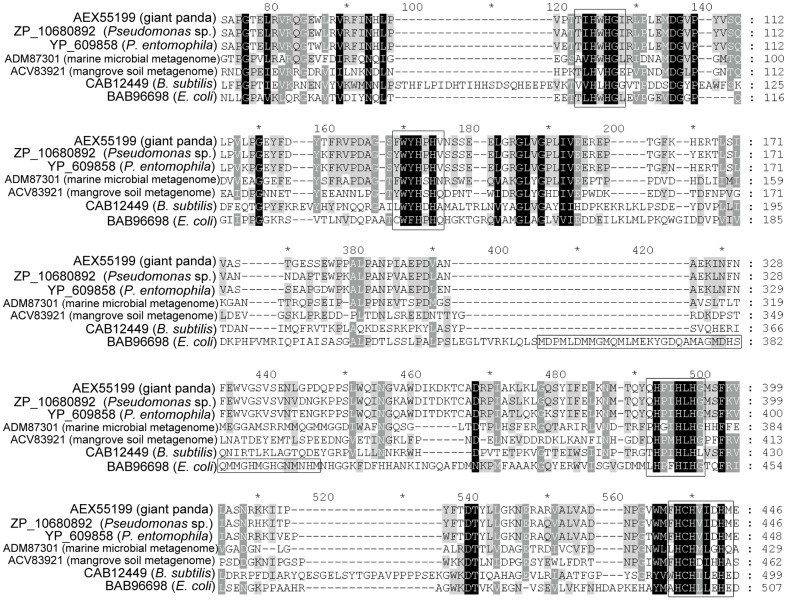
Multisequence alignment of Lac51 with multicopper oxidases in *Pseudomonas* spp. and some typical bacterial laccases. The protein sequences were retrieved from GenBank with the following accession numbers: the uncultured bacterium of this study (AEX55199), *Pseudomonas* sp. (ZP_10680892), *P. entomophila* (YP_609858), *B. subtilis* (CAB12449), *E. coli* (BAB96698), Lac15 from a marine microbial metagenome (ADM87301), and Lac591 from a mangrove soil metagenome (ACV83921). Sequence alignment was performed by Clustal ×2.0 and GENEDOC. Four histidine-rich copper binding domains were indicated by full-length vertical boxes and the methionine-rich region by horizontal box.

Sequence alignment showed that Lac51 is a laccase rather than a metallo-oxidase because it has the characteristics of typical laccases, such as, three conserved copper oxidase domains with the Pfam database accession numbers PF07732, PF00394, and PF07731. However, Lac51 does not contain a methionine-rich region, which is the 5th copper binding site in CueO from *E. coli* and may be a characteristic of metallo-oxidases (Figure 3) [35]. To confirm this hypothesis further, the Cu content of Lac51 was measured to exclude the possibility of the enzyme being a metallo-oxidase. Based on atomic absorption spectroscopy analysis, the molar ratio of copper atom to the active Lac51 was 3.9, which is very close to the 4 for typical laccases and indicates the four Cu ions incorporated into the active sites of the enzyme [31]. This result supports the previous hypothesis that Lac51 is a bacterial laccase rather than a metallo-oxidase.

To confirm biochemically that Lac51 is a laccase, it was recombinantly expressed with and without a TAT signal peptide in *E. coli* and assessed for the ability to oxidize syringaldazine, a typical laccase substrate. Using protein sequence analysis, Lac51 showed high identity with the multicopper oxidase from *Pseudomonas* sp., which indicates that the TAT signal peptide may not be recognized when using *E. coli* as the host. Therefore, when expressed with a signal peptide, the recombinant protein may have difficulty in folding properly and formed inclusion bodies. However, the recombinant protein without the TAT signal peptide can be expressed in a soluble form. Thus, the purified protein showed the highest oxidation activity towards the lignin-related phenolic compound syringaldazine (Table 2). All of these results indicate that Lac51 was a laccase.

**Table 2:** Relative activity of Lac51 towards lignin-related phenolic compounds at 38°C and optimum pH.

Substrate	Optimal pH	mU/mg	Relative Activity (%)
Syringaldazine	7.5	75.60	100
2,6-dimethoxyphenol	8.0	10.13	14
Gualacol	8.0	8.23	11
Ferulic acid	8.0	1.20	2
Sinapinic acid	8.0	0.77	1
Veratryl alcohol	8.0	0.53	0.7

### Lac51 oxidizes lignin-related phenolic compounds

**Figure 4:**
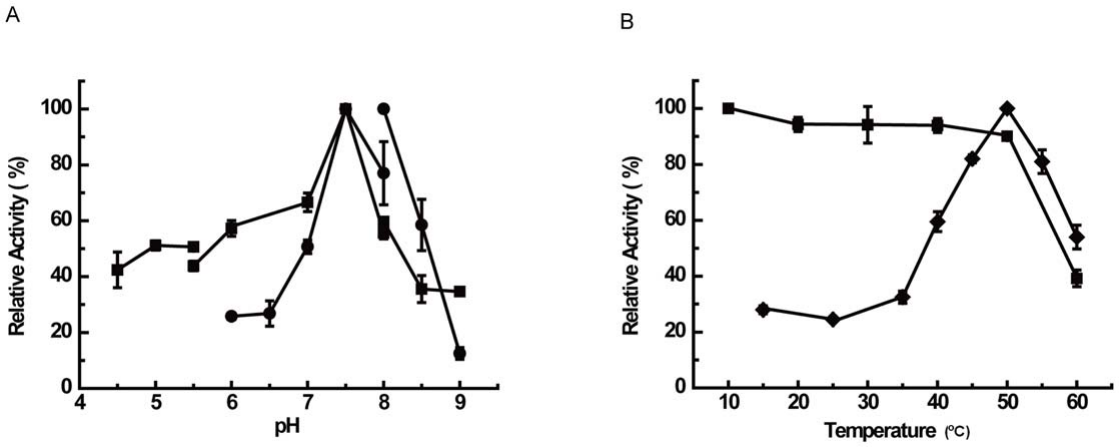
Biochemical activity of Lac51 towards syringaldazine. A Optimum pH (black circle) and pH stability (black square) of Lac51. Tests were performed at 50°C with syringaldazine as substrate. **B** Optimum temperature (black diamond) and themostability (black square). Lac51 was incubated in 50 mM Na_2_HPO_4_-KH_2_PO_4_ at pH7.5, with syringaldazine as substrate. The results were calculated from triplicate repeats of measurements. Error bars represent ±5 standard deviations.

Using syringaldazine as a substrate, biochemical characterization results showed that Lac51 oxidized syringaldazine under the optimal pH of 7.5 and temperature of 50°C (Figure 4). The highest activity exhibited 126 mU/mg. Lac51 was stable at pH ranging from 4.5 to 9.0 (Figure 4A). Thermostability tests showed that Lac51 was active at temperatures ranging from 15°C to 60°C and highly stable below 50°C (Figure 4B).

At 38°C and pH 7.5, which simulate the physiologic environment in the giant panda's intestine [36], Lac51 displayed approximately 60% of its maximum activity (Figure 4B), and it retained 51% of its activity after incubation at 38°C and pH 7.5 for 12 h.

The oxidation activity of Lac51 towards other lignin-derived phenolic compounds was also evaluated. The results showed that Lac51 oxidizes a variety of lignin-derived substrates, including 2,6-dimethoxyphenol, guaiacol, veratryl alcohol, sinapinic acid, and ferulic acid, at 38°C and pH 7.5–8.0 (Table 2). These phenolic compounds are generally regarded as lignin-derived moieties, so it is speculated that Lac51 may participate in lignin degradation, or is at least involved in the oxidation of phenol compounds.

### Lac51 is involved in lignin oxidation

**Figure 5:**
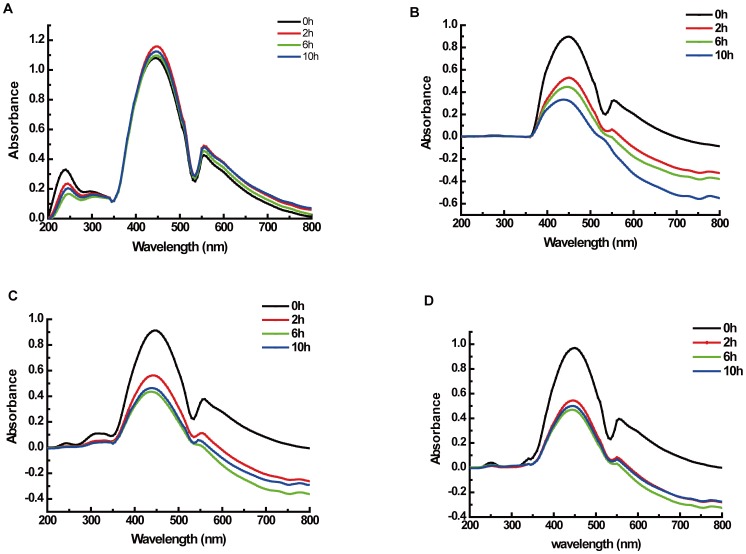
Enzymatic treatment of Lac51 towards lignin tested by full wavelength scanning. Absorbance of lignin by full wavelength scanning from 200 nm to 800 nm (**A**). Changes in absorbance of lignin treated with Lac51 for 2, 6, and 10 h in the presence of ABTS (B), syringic acid (C) and ferulic acid (D) as mediators.

To confirm further that Lac51 participates in the lignin oxidation process, full wavelength scanning was employed to investigate the effects of Lac51 on lignin with or without a mediator. The results showed that the absorbance of lignin in Na_2_HPO_4_–KH_2_PO_4_ buffer did not change even after 10 h of incubation at 37°C (Figure 5A). Treating lignin with Lac51 alone (without mediator) at 37°C and pH 7.5 did not affect absorbance. However, in the presence of mediators, the absorbance changed. Among the mediators, ABTS, syringic acid, and ferulic acid promoted lignin catalysis by Lac51, as shown by the time-dependent decrease in absorbance (Figures 5B–5D). During the 10 h incubation, the absorbance at 445 nm declined dramatically within the first two hours. Then, the absorbance ultimately decreased to 36% for ABTS, 51% for syringic acid, and 51% for ferulic acid, which indicates that Lac51 may be involved in lignin oxidation.

## Discussion

We profiled the microbial flora that inhabits the giant panda gut by analyzing 16S rRNA gene sequences to investigate the possible existence of lignin-degrading related bacteria. A metagenomic approach was also employed to identify the lignolysis-related enzymes in the gut microbiome.

Recovery and analysis of the 16S rRNA gene provide an effective way of investigating gut microbial flora independent of pure cultures [37]. In this study, the bacterial community derived from the giant panda gut was investigated by constructing a 16S rRNA gene library with 90.3% coverage and grouping into 14 phylotypes (Table 1). Similar to our study, large-scale analysis of the 16S rRNA gene sequences from the digestive system of giant pandas revealed a low species richness in the microbiome at 4 OTUs to 25 OTUs, which were already saturated based on the rarefaction analysis [4]. Within the members of the *Ursidae* family, which includes carnivores, herbivores, and omnivores, the number of OTUs ranged from 14 to 34 [38]. The number of OTUs observed in the feces samples from giant pandas and polar bears was within this range, which was nearly saturated as revealed by the asymptotic nature of the rarefaction curves (Figure 1) [4]. However, the species richness in the gut of giant pandas was lower than that of herbivores, of which the number of OTUs observed was far from saturation by rarefaction analysis (Figure 1) [39], [40]. Therefore, the low fecal microbial diversity may be characteristic of giant pandas, which may result from their phylogeny and special bamboo diet [4], [41].

All of the 16S rRNA gene sequences obtained from the giant panda intestine microbiome belonged to the *Alphaproteobacteria*, *Gammaproteobacteria*, and *Clostridia* classes. Of the phylotypes in class *Clostridia*, four phylotypes were closest to *Clostridium* group I, which contains the taxa capable of digesting cellulose and reportedly plays an important role in helping the giant panda digest cellulose and hemicellulose of bamboos [4], [36]. Similar to the lignin in trees, the lignin in bamboo forms a matrix with cellulose and hemicellulose, which hampers the digestion of these two carbon sources by organisms. Aside from fungi, bacterial strains capable of degrading lignin have been reported. However, this area is still less extensively studied [6], [17], [42], [43]. To date, the majority of identified bacterial lignin degraders have been classified into three classes, the *Actinomycetes*, *Alphaproteobacteria*, and *Gammaproteobacteria* [17], [18], such as *Sphingomonas* sp. from *Alphaproteobacteria* [44]–[46]; *Pseudomonas* sp. from *Gammaproteobacteria* [47], [48]; and *Rhodococcus*, *Nocardia*, and *Streptomyces* from *Actinomycetes* [6], [49]. In the current work, most of the 16S rRNA gene sequences detected belonged to *Gammaproteobacteria*. Furthermore, two phylotypes were affiliated with mangrove forest bacteria and *P. putida*, which are related to lignin decomposers. Thus, the gut microbes may help giant pandas facilitate lignin digestion during the utilization of cellulose and hemicellulose.

Laccases, regarded as lignolysis-related enzymes, were found in the gut microbes of giant pandas in this study. Two laccase genes were obtained from the metagenomic library and shared high sequence identities with putative multicopper oxidases of *Pseudomonas* spp., such as *Pseudomonas* sp. GM30 (88%), *P. entomophila* (80%), and *P. putida* KT2440 (80%). Genome analysis of *P. entomophila* revealed that most of the catabolic genes were closely related to *P. putida* KT2440, which is the plasmid-free derivative of *P. putida* mt-2 and is found to possess metabolic pathways for the transformation of a variety of aromatic derivatives of lignin arising from the decomposition of plant materials [50], [51]. Furthermore, recent bioinformatics analysis showed that most bacterial laccases are possibly extracellular enzymes, in contrast to the current view that they are intracellularly located [18], [52]. In accordance with this viewpoint, a TAT signal peptide was found at the N-terminus of Lac51. The TAT signal peptide allows the exportation of fully folded proteins from the cytoplasm across the inner cytoplasmic membrane. The presence of a TAT signal peptide in Lac51 suggests that the enzyme may take effect after its export out of the cytoplasm.

Laccases enhance microbial resistance to phenolic compounds generated from the partial breakdown of lignin by decreasing their redox potential and, to some extent, facilitate lignin degradation [53]–[55]. During lignin degradation, toxic phenolic compounds are produced, such as aromatic acids, alcohols, and aldehydes [56], which may inhibit the growth and viability of microorganisms [57]. Laccases oxidize phenolic compounds by generating unstable phenoxy radicals that lead to polymerization into less toxic aromatic compounds [55], [58]. The discovery of laccase activity in giant panda intestines indicates its possible involvement in the detoxification process during lignin degradation in the gut environment of giant pandas. On the other hand, natural phenolic compounds, especially the lignin degradation products, are considered candidates for mediating laccase-catalyzing reactions, such as lignin depolymerization [59]–[61]. In the presence of the two lignin-derived phenolic compounds ferulic acid and syringic acid as the mediators, Lac51 has been shown to catalyze lignin effectively. The laccase activity in the giant panda gut indicated that the bacteria might produce lignolysis-related enzymes, such as Lac51, which may play positive roles in facilitating the breakdown of bamboo lignin in the gut environment of giant pandas. Although further lignin degradation studies are necessary, the findings reported here indicate that laccases may be involved in the detoxification of lignin-related phenolic compounds and they act on lignin to some extent in the giant panda gut.

Along with the discovery of putative cellulose-metabolizing enzymes [4], the laccases produced by the microorganisms in the giant panda intestines explain how giant pandas partially digest bamboo lignocellulose despite the lack of genes that encode lignocellulose-degrading enzymes in their genome. Furthermore, a better understanding of bamboo lignocellulose digestion may be helpful for improving the bamboo digestion by giant pandas and consequently contribute to the better protection of this endangered species.

## Supporting Information

Figure S1 **Phylogenetic tree of the intestinal bacteria of giant panda.** Near-full-length 16S rRNA gene sequences were aligned to their closest neighbors in the NCBI database. The tree was inferred based on the neighbor-joining algorithm, and bootstrap values shown at the branches are based on 1000 replicates.
(TIF)
