# Supplementary figures and images for: Evidence for Lignin Oxidation by the Giant Panda Fecal Microbiome
Source: PLoS One. 2012 Nov 28;7(11):e50312. doi: 10.1371/journal.pone.0050312 (PMC3508987; doi:10.1371/journal.pone.0050312)

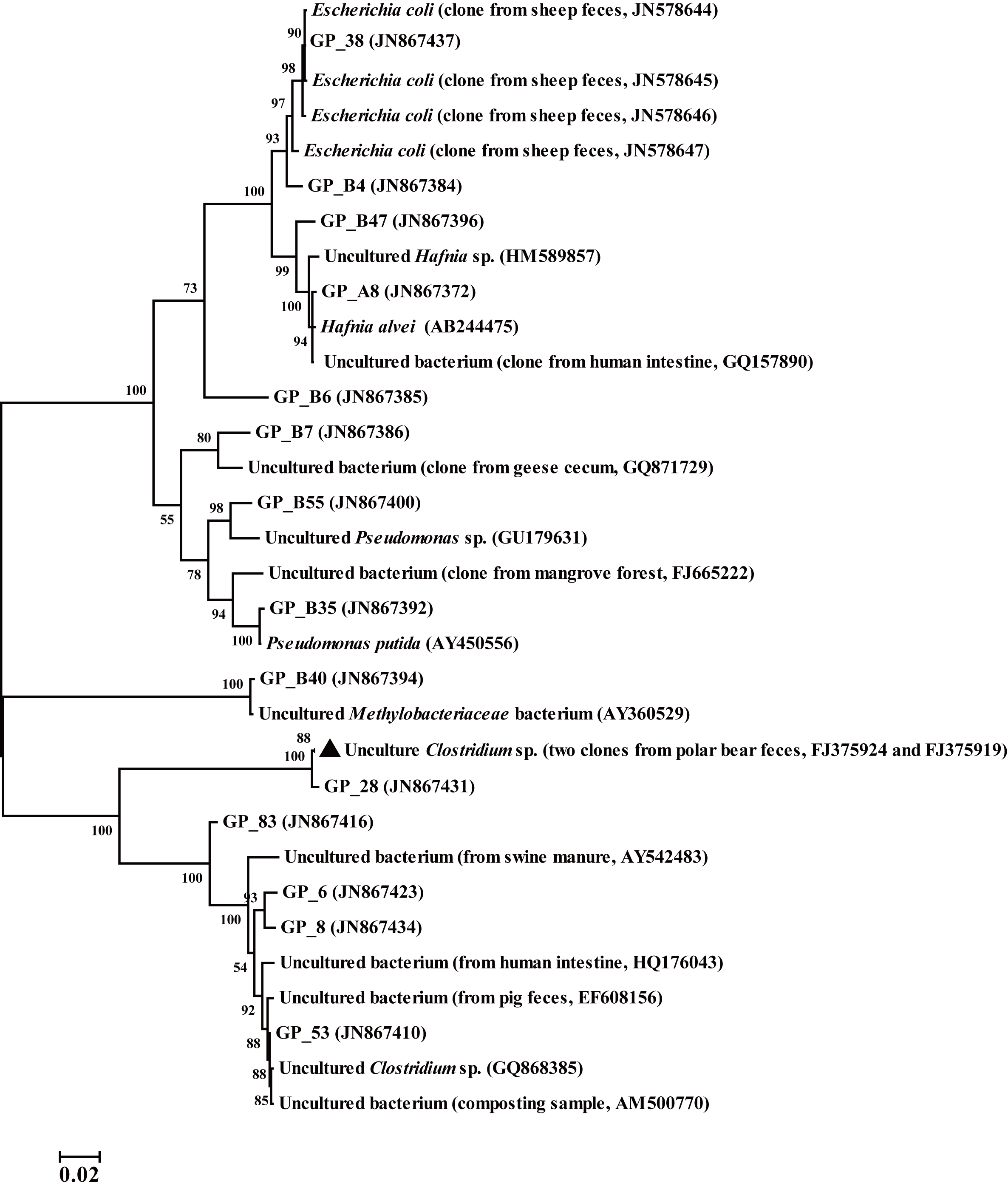

Supplement: Figure S1 — Phylogenetic tree of the intestinal bacteria of giant panda. Near-full-length 16S rRNA gene sequences were aligned to their closest neighbors in the NCBI database. The tree was inferred based on the neighbor-joining algorithm, and bootstrap values shown at the branches are based on 1000 replicates. (TIF) [file pone.0050312.s001.tif]
